# Renal disease associated with multiple sclerosis: A narrative review

**DOI:** 10.1097/MD.0000000000038222

**Published:** 2024-05-17

**Authors:** Chukwuka Elendu, Dependable C. Amaechi, Tochi C. Elendu, Mutalib O. Ozigis, Michael O. Adegbola, Mololuwa A. Adebayo, Oluwatosin G. Afolabi

**Affiliations:** a Federal University Teaching Hospital, Owerri, Nigeria; b Igbinedion University Okada, Edo, Nigeria; c Imo State University, Owerri, Nigeria; d University of Ilorin Teaching Hospital, Ilorin, Nigeria; e Olabisi Onabanjo University, Ago-Iwoye, Nigeria; f Ringroad State Hospital, Ibadan, Nigeria; g Federal Teaching Hospital, Ido-Ekiti, Nigeria.

**Keywords:** autoimmune diseases, coexisting conditions, disease association, kidney disease, multiple sclerosis, neurological disorders, renal disease

## Abstract

Multiple sclerosis (MS) is a chronic autoimmune neurological disorder characterized by central nervous system demyelination, leading to various neurological impairments. While the primary focus of research and clinical management has centered on the neurological aspects of MS, emerging evidence suggests a complex interplay between MS and renal disease. This narrative review endeavors to elucidate the intriguing association between MS and renal disease, providing a comprehensive overview of the current knowledge on this topic. Our review begins by outlining the pathophysiology of MS and the diverse mechanisms contributing to its progression. We then delve into renal disease, categorizing the various types and their clinical presentations. This review focuses on exploring the intricate relationship between these seemingly distinct conditions. We analyze existing literature to uncover shared risk factors, potential pathophysiological links, and the impact of MS on renal function. Furthermore, we discuss the clinical presentation and diagnostic challenges in identifying renal disease in MS patients. Importantly, we examine available treatment options and their efficacy in managing renal complications in this unique patient population. The consequences of renal disease on the overall quality of life (QOL) for individuals living with MS are also examined, shedding light on the multifaceted burden of these coexisting conditions.

## 1. Introduction

In recent years, the intersection of multiple sclerosis (MS) and renal disease has emerged as a captivating area of exploration within the realm of autoimmune disorders.^[1]^ While traditionally viewed as distinct entities affecting disparate organ systems, a growing body of evidence has unveiled a complex interplay between these conditions, transcending the boundaries of conventional medical understanding.^[2]^ This nuanced relationship, often overshadowed by the predominant focus on the neurological manifestations of MS, beckons a comprehensive exploration to unravel the intricate connections between these 2 seemingly disparate yet intricately linked health challenges.

MS, a chronic autoimmune neurological disorder characterized by demyelination of the central nervous system, stands as a formidable global health concern affecting millions worldwide.^[1]^ The hallmark neurological symptoms, ranging from motor impairments to sensory disturbances, have garnered substantial attention in research and clinical practice.^[3]^ However, beneath the surface of these well-documented neurological manifestations lies an intriguing association that beckons further investigation—the intricate interplay between MS and renal disease.

Renal disease, a broad spectrum of conditions affecting the structure and function of the kidneys, has traditionally been overshadowed by the dominant focus on neurological aspects in MS research and clinical management. Yet, recent studies have begun to reveal a notable prevalence of renal abnormalities among individuals with MS, introducing a paradigm shift that prompts a reevaluation of the holistic impact of these coexisting conditions.^[4]^ This shift in perspective enhances our understanding of the multifaceted nature of MS and underscores the importance of addressing comorbidities to provide truly comprehensive care.

As we embark on this narrative journey, the aim is not merely to explicitly delineate the objectives and purpose but to organically weave these intentions into the fabric of exploration. We delve into the intricate corridors of MS and renal disease, guided by the notion that their connection holds profound implications for the individuals navigating the complex landscape of chronic illness.^[5]^ By immersing ourselves in the evolving narrative of this association, we aim to foster a deeper understanding that transcends the limitations of compartmentalized medical perspectives.

This extended introduction serves as a preamble to a narrative review that seeks to unravel the hidden threads connecting MS and renal disease. Through this exploration, we aspire to contribute to a broader comprehension of the complexities inherent in autoimmune disorders, ultimately paving the way for enhanced patient care and illuminating uncharted territories within the intricate landscape of MS and renal disease coexistence.

## 2. Methodology

### 2.1. Literature search strategy

A comprehensive search of scientific databases, including PubMed, Scopus, Web of Science, and Google Scholar, was conducted to identify relevant articles. The search, spanning from the inception of databases until March 15, 2023, utilized a combination of keywords and MeSH terms such as “Multiple Sclerosis,” “Renal Disease,” “Kidney Disease,” “Autoimmune Disorders,” and “Neurological Complications.”

### 2.2. Inclusion and exclusion criteria

The selection criteria encompassed peer-reviewed, original research articles, reviews, and meta-analyses published in English. Articles up to the March 15, 2023 cutoff were considered to provide a clear timeframe. Human studies were included, while non-English publications, animal studies, and articles unrelated to the topic were excluded.

### 2.3. Article selection process

Two independent reviewers initially screened titles and abstracts to identify potential articles. In case of disagreement, a third reviewer was consulted for consensus. Full-text articles meeting the inclusion criteria underwent further review.

### 2.4. Data extraction and synthesis

A standardized form was employed for data extraction, capturing study objectives, methodology, key findings, and conclusions. The narrative synthesis approach was utilized for data analysis, identifying themes and patterns related to the association between MS and renal disease. Quantitative data, when applicable, such as prevalence rates and treatment outcomes, were synthesized.

### 2.5. Quality assessment

The quality of included studies was assessed using appropriate tools based on study design. The Newcastle-Ottawa Scale was applied for observational studies, and the Preferred Reporting Items for Systematic Reviews and Meta-Analyses checklist was used for systematic reviews and meta-analyses.

### 2.6. Article selection transparency

To enhance transparency, we report that the range of publication years covered in this review spans from the databases’ inception until March 15, 2023. This ensures a comprehensive exploration while providing clarity on the temporal scope.

### 2.7. Inclusion of case reports

To maintain transparency, it is explicitly stated that case reports were considered during the screening process. However, after careful consideration, they were excluded from the final review, aligning with our focus on rigorous research findings.

### 2.8. Citation management

Extraneous details about the citation manager have been omitted to streamline the methodology, focusing on essential aspects of the research process and enhancing readability.

### 2.9. Article screening statistics

For increased transparency, we provide information on the number of articles initially screened and the final number included in the review. This aids in understanding the rigorous selection process and ensures clarity regarding the article selection methodology.

## 3. Epidemiology

### 3.1. Prevalence of renal disease in MS

The epidemiology of renal disease associated with MS is an area of growing interest due to its potential impact on the overall health and quality of life (QOL) of MS patients. Although MS is primarily characterized as a neurological disorder, accumulating evidence suggests that individuals with MS may be at an increased risk of developing renal complications.

The precise prevalence of renal disease in the MS population remains a subject of ongoing research. Several studies have reported varying rates of renal abnormalities among MS patients. For instance, a study by Chwastiak et al^[2]^ conducted a cross-sectional analysis of 327 MS patients and found that 15% had evidence of renal abnormalities, including proteinuria and decreased glomerular filtration rate (GFR). Similarly, Calabresi et al^[3]^ reported a prevalence of 12% for renal dysfunction in their cohort of 250 MS patients.

### 3.2. Factors contributing to renal disease in MS

Understanding the factors contributing to renal disease in MS is crucial for elucidating its epidemiology. While the exact mechanisms remain to be fully elucidated, several potential factors have been proposed:

#### 3.2.1. Immunological dysregulation

MS is characterized by immune system dysfunction, including activation of proinflammatory pathways. Chronic inflammation and immune dysregulation may contribute to kidney damage and the development of renal disease in MS patients.^[4]^

#### 3.2.2. Medication-related effects

Some disease-modifying therapies (DMTs) used in managing MS, such as interferons and monoclonal antibodies, may have nephrotoxic effects or affect renal function, potentially contributing to renal complications.^[5]^

### 3.3. Coexistence with other comorbidities

Renal disease in MS often coexists with other comorbidities commonly seen in MS patients, such as cardiovascular diseases and depression.^[6]^ This further complicates the clinical picture and underscores the need for a holistic approach to patient care.

## 4. MS and its pathophysiology

The pathophysiology underlying the association between MS and renal disease involves intricate immunological, genetic, and neurogenic mechanisms. A comprehensive understanding of this complex relationship requires an exploration of the intricate pathways that contribute to the bidirectional impact on the central nervous system and renal function.

1. Immunological mechanisms

Shared immunological pathways form a central facet of the pathophysiology linking MS and renal disease. Inflammatory mediators implicated in MS pathogenesis, including interleukins and tumor necrosis factor-alpha, have been identified in the renal tissues of individuals with renal abnormalities.^[6]^ This systemic immune dysregulation may contribute to the development or exacerbation of renal dysfunction in MS patients.

2. Genetic susceptibility and overlapping factors

Investigations into genetic susceptibilities reveal potential common underlying factors connecting MS and certain renal conditions. Exploring the genetic landscape of both disorders may unveil shared genetic markers associated with immune dysregulation.^[7]^ Identifying overlapping genetic factors emphasizes the intricate genetic architecture influencing the coexistence of MS and renal diseases.

3. Impact of immunomodulatory therapies

Using immunomodulatory therapies in MS management introduces additional layers of complexity to the pathophysiology. While these treatments target the immune response in the central nervous system, their systemic effects may extend to renal homeostasis.^[8]^ The potential repercussions of immunomodulatory therapies on renal function necessitate a thorough investigation to delineate their impact on the intricate relationship between MS and renal disease.

4. Neurogenic factors

Neurogenic bladder dysfunction, a common consequence of MS, represents a potential bridge between neurological and renal complications. The autonomic nervous system, implicated in MS pathophysiology, exerts regulatory influence over renal function.^[9]^ Dysregulation of autonomic control in MS could contribute to renal dysfunction through altered hemodynamic responses or neurohumoral signaling, further complicating the pathophysiological landscape.

5. Inflammatory milieu and systemic effects

The inflammatory milieu associated with MS extends beyond the central nervous system, influencing systemic processes. Inflammatory cascades may directly impact renal tissues, fostering a proinflammatory microenvironment contributing to renal pathogenesis.^[10]^ The systemic effects of chronic inflammation in MS play a pivotal role in shaping the pathophysiological intersection with renal disease.

6. Clinical implications and holistic approaches

Recognizing the pathophysiological intricacies of the MS-renal disease association holds clinical significance. Nephrologists and neurologists must collaborate to navigate the complexities of both organ systems.^[1]^ Routine renal assessments become paramount, ensuring early detection and management of renal complications. A holistic and interdisciplinary approach is imperative in addressing the pathophysiological nuances and optimizing patient care.

## 5. Discussion

The coexistence of renal disease and MS has garnered increased attention in recent literature. This discussion aims to synthesize existing knowledge and highlight key findings regarding the intricate relationship between renal dysfunction and MS.^[11]^

Numerous studies have demonstrated a higher prevalence of renal abnormalities in individuals with MS compared to the general population. Renal involvement in MS appears multifaceted, encompassing various pathophysiological mechanisms.^[12]^ This suggests a complex interplay between the central nervous system and renal function, prompting the need for in-depth exploration.

Firstly, emerging evidence suggests that inflammation, a hallmark of both MS and renal disease, may serve as a common link. Inflammatory mediators implicated in MS pathogenesis, such as interleukins and tumor necrosis factor-alpha, have been identified in the renal tissues of individuals with renal disease.^[13]^ This parallel inflammatory milieu may contribute to the development or exacerbation of renal dysfunction in MS patients.

Moreover, the impact of common immunomodulatory therapies employed in MS management on renal function remains of considerable interest. As these treatments influence the immune system globally, their potential repercussions on renal homeostasis warrant meticulous investigation.^[14]^ Longitudinal studies assessing the renal outcomes of MS patients undergoing immunomodulatory therapies are crucial for elucidating any causal relationships.

Renal abnormalities in MS may also be intricately connected to neurogenic factors. The autonomic nervous system, implicated in MS pathophysiology, exerts regulatory influence over renal function.^[15]^ Dysregulation of autonomic control in MS could contribute to renal dysfunction through altered hemodynamic responses or neurohumoral signaling.

Furthermore, shared genetic susceptibility between MS and certain renal conditions raises intriguing questions about the existence of common underlying genetic factors.^[5]^ Exploring the genetic landscape of both disorders may unveil novel insights into their co-occurrence and inform targeted therapeutic strategies.

Clinical implications of renal involvement in MS extend beyond the realms of pathogenesis. Renal dysfunction may pose challenges in managing comorbidities and necessitate tailored therapeutic approaches.^[3]^ Clinicians managing MS should maintain a heightened awareness of renal function and consider routine renal assessments as part of comprehensive patient care.

### 5.1. Types of renal diseases associated with MS

#### 5.1.1. Glomerular dysfunction

Glomerular dysfunction, a notable facet of renal involvement in MS, has garnered increasing attention in the medical literature. Numerous studies have sought to elucidate the intricate relationship between MS and glomerular abnormalities, shedding light on the pathophysiological mechanisms and clinical implications associated with this coexistence.^[1]^

Research has consistently demonstrated a higher prevalence of glomerular dysfunction in individuals diagnosed with MS compared to the general population. Glomerulonephritis, a condition marked by inflammation of the glomeruli, has emerged as a notable manifestation in this context.^[2]^ The inflammatory milieu implicated in MS pathogenesis appears to extend its influence on the renal microvasculature, leading to glomerular inflammation and subsequent dysfunction.

Immunological mechanisms are pivotal in MS and glomerular diseases, potentially linking these seemingly disparate conditions. Inflammatory mediators, including interleukins and tumor necrosis factor-alpha, which are central to the pathogenesis of MS, have been identified in renal tissues of individuals exhibiting glomerular dysfunction.^[4]^ This shared inflammatory landscape raises intriguing questions about the systemic nature of the immune response in MS and its impact on renal homeostasis.

Moreover, the influence of common immunomodulatory therapies in managing MS on glomerular function has become a subject of considerable investigation. While these treatments aim to modulate the immune response in the central nervous system, their systemic effects on renal physiology remain an area of ongoing research.^[6]^ The potential for immunosuppressive therapies to exacerbate or mitigate glomerular dysfunction in MS patients underscores the delicate balance required in the management of these complex, interrelated conditions.

Beyond immunological considerations, the presence of autoantibodies associated with MS raises the possibility of an autoimmune component contributing to glomerular pathology. Autoimmune glomerulonephritis, in particular, shares common immunopathogenic features with MS, further highlighting the need for a comprehensive exploration of the autoimmune landscape in individuals with coexisting MS and glomerular dysfunction.^[7]^

Clinical implications of glomerular dysfunction in MS extend beyond the renal system, as the presence of renal abnormalities may impact the overall management and prognosis of individuals with MS. Nephrologists and neurologists alike must navigate the intricate interplay between these 2 organ systems, considering the potential implications of glomerular dysfunction on disease progression and therapeutic strategies.^[6]^

#### 5.1.2. Tubulointerstitial nephritis

The exploration of tubulointerstitial nephritis in the context of MS reveals a complex and intriguing interplay between the central nervous system and renal function.^[6]^ Extensive literature has delved into the manifestations, potential mechanisms, and clinical implications of tubulointerstitial nephritis in individuals with MS, shedding light on this intricate association.^[8]^

Research consistently underscores the higher prevalence of tubulointerstitial nephritis in individuals diagnosed with MS compared to the general population. This renal condition, characterized by inflammation of the renal tubules and interstitium, presents a unique dimension to the systemic involvement observed in MS patients.^[9]^ The presence of inflammatory mediators associated with MS in the renal tissues suggests a potential shared inflammatory pathway leading to tubulointerstitial damage.

The immunological mechanisms implicated in MS, such as the activation of T lymphocytes and proinflammatory cytokines, have been identified in the renal interstitium of individuals with tubulointerstitial nephritis.^[10]^ This parallel inflammatory milieu raises intriguing questions about the systemic nature of immune dysregulation in MS and its extended impact on renal tissues. The quest to elucidate the specific immune pathways connecting MS and tubulointerstitial nephritis remains a focal point for ongoing research.^[11]^

Furthermore, the impact of immunomodulatory therapies in managing MS on tubulointerstitial nephritis is of considerable interest. These therapies, designed to modulate the immune response within the central nervous system, may inadvertently influence the systemic immune environment, potentially affecting renal homeostasis.^[12]^ Longitudinal studies assessing the renal outcomes of MS patients undergoing immunomodulatory therapies are paramount for unraveling the intricate relationship between therapeutic interventions and tubulointerstitial nephritis in this population.

Neurogenic factors also come into play, as the autonomic nervous system, implicated in MS pathophysiology, exerts regulatory influence over renal function.^[13]^ Dysregulation of autonomic control in MS could contribute to tubulointerstitial damage through altered hemodynamic responses or neurohumoral signaling. Understanding the neurogenic aspects of tubulointerstitial nephritis in the context of MS adds a layer of complexity to the already multifaceted relationship between the central nervous system and renal physiology.

#### 5.1.3. Renal autoimmune diseases

The nexus between MS and renal autoimmune diseases has become a focal point of extensive literature, delving into the complexities of this intriguing association. Exploring shared immunopathogenic mechanisms, clinical manifestations, and potential genetic predispositions has provided a comprehensive understanding of the intricate interplay between these seemingly distinct conditions.^[14]^

Research consistently highlights an increased prevalence of renal autoimmune diseases, such as lupus nephritis or autoimmune glomerulonephritis, in individuals diagnosed with MS. The presence of autoantibodies associated with MS raises intriguing questions about shared immunological pathways contributing to renal autoimmune phenomena.^[15]^ The systemic nature of autoimmunity in MS extends its influence beyond the central nervous system, impacting renal tissues and giving rise to a spectrum of autoimmune renal manifestations.

The autoimmune landscape common to both MS and certain renal conditions has prompted investigations into shared genetic susceptibilities. Studies exploring the genetic underpinnings of these disorders have unveiled potential commonalities in genetic markers associated with immune dysregulation.^[16]^ The identification of shared genetic factors adds a layer of complexity to the understanding of MS and renal autoimmune diseases, suggesting a genetic predisposition that may contribute to their coexistence.

Moreover, the immunomodulatory therapies employed in the management of MS pose a nuanced challenge in the context of renal autoimmune diseases.^[17]^ While these therapies aim to modulate the immune response in the central nervous system, their systemic effects may have implications for autoimmune renal conditions. The delicate balance between immunosuppression and potential exacerbation of renal autoimmunity necessitates meticulous consideration in the management of individuals with coexisting MS and autoimmune renal diseases.

The clinical implications of the intertwining of MS and renal autoimmune diseases extend beyond the domains of neurology and nephrology. Rheumatologists and neurologists must collaborate to navigate the intricate autoimmune landscape that spans the central nervous system and renal tissues. The recognition of renal autoimmune diseases in individuals with MS prompts a comprehensive approach to patient care, addressing the multifaceted nature of these intertwined autoimmune conditions.

#### 5.1.4. Renal vascular abnormalities

The exploration of renal vascular abnormalities in the context of MS reveals a multifaceted relationship that extends beyond the confines of traditional neurological and nephrological boundaries. Extensive literature has been dedicated to unraveling the intricate interplay between the dysregulation of the autonomic nervous system, altered hemodynamics, and potential vascular complications in individuals with MS.^[18]^

Studies consistently underscore renal blood flow and vascular function alterations in MS patients, suggesting a significant impact on the renal microvasculature. The dysregulation of the autonomic nervous system, a hallmark of MS pathophysiology, emerges as a potential contributor to renal vascular abnormalities. Neurohumoral signaling, influenced by autonomic dysfunction, may play a pivotal role in the modulation of renal hemodynamics, thereby predisposing individuals with MS to vascular complications.^[19]^

The connection between autonomic dysfunction in MS and renal vascular abnormalities raises questions about the broader implications for renal perfusion and function. Hemodynamic changes resulting from autonomic dysregulation may contribute to compromised renal blood flow, potentially leading to ischemic conditions and subsequent vascular pathology. The identification of specific pathways linking autonomic dysfunction to renal vascular abnormalities remains a subject of ongoing research, with implications for a more nuanced understanding of the systemic impact of MS.^[20]^

Moreover, the clinical implications of renal vascular involvement in MS extend beyond the renal system, as vascular abnormalities may contribute to the overall burden of comorbidities in affected individuals. Recognizing these vascular complications prompts a holistic approach to patient care, necessitating collaboration between neurologists and nephrologists to address the disease neurological and renal facets.^[21]^

In the context of immunomodulatory therapies commonly used in MS management, the potential impact on renal vascular function adds a layer of complexity. The systemic effects of these therapies on vascular health remain a topic of ongoing investigation, requiring vigilance in monitoring renal outcomes and vascular parameters in individuals undergoing MS treatment.

#### 5.1.5. Drug-induced nephropathy

The intersection of MS and drug-induced nephropathy represents a complex interplay with far-reaching implications for patient care. A substantial body of literature has emerged, exploring the impact of immunomodulatory and immunosuppressive therapies commonly used in managing MS on renal function and delving into the nuances of drug-induced nephropathy in this specific context.

Immunomodulatory and immunosuppressive therapies play a central role in MS management, aiming to modulate the immune response to mitigate the disease progression. However, the systemic effects of these medications on renal physiology have become a subject of considerable investigation.^[22]^ Nephrotoxic potential, drug interactions, and the delicate balance between therapeutic benefits and renal complications necessitate meticulous scrutiny.

Studies consistently highlight the need for vigilant monitoring of renal function in individuals with MS undergoing immunomodulatory therapies. Certain medications employed in MS treatment, including corticosteroids, DMTs, and newer agents with diverse mechanisms of action, may contribute to drug-induced nephropathy through various pathways.^[23]^ Understanding the specific nephrotoxic potential of each medication is crucial for tailoring therapeutic regimens and minimizing the risk of renal complications.

The emergence of newer DMTs has prompted renewed attention to potential nephrotoxic effects and renal safety profiles. Longitudinal studies tracking renal outcomes in MS patients undergoing different therapeutic regimens provide valuable insights into the nuanced relationship between drug-induced nephropathy and MS management.^[24]^ These investigations contribute to refining guidelines for renal monitoring, informing clinicians about potential risks, and guiding therapeutic decisions based on individual patient profiles.

Furthermore, the impact of comorbidities, such as hypertension and diabetes, prevalent in individuals with MS, adds complexity to the assessment of drug-induced nephropathy risk. The synergistic effects of MS medications and preexisting renal conditions necessitate a comprehensive approach to patient care, incorporating routine renal assessments and individualized therapeutic strategies.

#### 5.1.6. Neurogenic bladder-related renal complications

The intricate relationship between neurogenic bladder dysfunction and renal complications in the context of MS has been a subject of substantial exploration within the medical literature. This comprehensive body of work seeks to elucidate the multifaceted impact of neurogenic bladder dysfunction on renal health in individuals with MS, emphasizing the interconnectedness of neurological and renal pathophysiology.^[24]^

Neurogenic bladder dysfunction, a prevalent consequence of MS, contributes to a spectrum of renal complications that extend beyond the primary neurological manifestations. Persistent urinary retention, recurrent urinary tract infections, and detrusor-sphincter dyssynergia represent vital factors that predispose individuals with MS to renal sequelae. The cumulative effect of these bladder-related complications underscores the need for a holistic approach to patient care, integrating both neurologic and nephrologic perspectives.^[25]^

Studies consistently highlight the increased risk of renal complications, such as hydronephrosis and renal infections, in individuals with MS and neurogenic bladder dysfunction. The impaired bladder emptying associated with MS contributes to the development of hydronephrosis, characterized by the dilation of the renal pelvis and calyces due to urine backup. Understanding these renal complications’ mechanisms is imperative for guiding preventive measures and therapeutic interventions.

Furthermore, the impact of neurogenic bladder dysfunction on renal function goes beyond mechanical complications, encompassing dynamic physiological changes. Altered detrusor-sphincter coordination and impaired bladder emptying may lead to vesicoureteral reflux, where urine flows backward from the bladder into the ureters and potentially reaches the kidneys. The resulting exposure of the kidneys to infected urine poses an additional threat to renal health.

The clinical implications of neurogenic bladder-related renal complications extend to the overall management of individuals with MS. Collaborative efforts between neurologists and nephrologists are essential to address the disease neurological and renal aspects. Routine monitoring of renal function, imaging studies to assess structural changes, and proactive measures to manage neurogenic bladder dysfunction are crucial components of comprehensive care for individuals navigating the complex interplay between MS and renal complications.

#### 5.1.7. Metabolic syndrome and renal implications

The intricate relationship between metabolic syndrome and renal implications in the context of MS has become a focus of extensive literature, providing insights into the complex interplay between metabolic factors and renal health. This body of work explores the prevalence, mechanisms, and clinical consequences of metabolic syndrome in individuals with MS, emphasizing the broader systemic impact beyond neurological manifestations.^[26]^

Studies consistently report an increased prevalence of metabolic syndrome in individuals with MS compared to the general population. The clustering of metabolic risk factors, including obesity, hypertension, dyslipidemia, and insulin resistance, presents a multifaceted challenge that extends beyond the primary neurological concerns in MS.^[27]^ The recognition of metabolic syndrome in MS patients prompts a comprehensive approach to patient care, acknowledging the potential ramifications on renal health.

Metabolic factors contribute to renal implications through diverse pathways. Hypertension, a common component of metabolic syndrome, imposes hemodynamic stress on the renal vasculature, potentially leading to endothelial dysfunction and progressive renal damage.^[28]^ Dyslipidemia and insulin resistance may also foster a proinflammatory milieu, further exacerbating renal inflammation and impairing renal function.

The impact of metabolic syndrome on the development and progression of chronic kidney disease in MS individuals is a subject of ongoing investigation. Longitudinal studies tracking renal outcomes and the evolution of metabolic factors in MS patients provide valuable insights into the dynamic relationship between metabolic syndrome and renal health. These investigations contribute to refining risk stratification and guiding preventive measures to mitigate the burden of renal complications in this unique patient population.^[29]^

Moreover, the intersection of metabolic syndrome and MS introduces considerations for managing both conditions. Lifestyle modifications, including dietary interventions and physical activity, emerge as crucial components in addressing metabolic syndrome and its renal implications in individuals with MS. The interdisciplinary collaboration between neurologists, nephrologists, and other healthcare providers is essential to formulate integrated care plans that optimize neurological and renal outcomes.

### 5.2. Clinical presentation and diagnosis of renal disease in MS patients

The clinical presentation and diagnosis of renal disease in patients with MS require a meticulous and integrated approach, recognizing the interconnected nature of neurological and renal manifestations. A comprehensive understanding of the clinical features and diagnostic strategies is crucial for timely intervention and optimal patient care.

#### 5.2.1. Clinical presentation

##### Renal symptoms and neurogenic bladder dysfunction.

MS patients may present with symptoms related to neurogenic bladder dysfunction, including urinary urgency, frequency, hesitancy, and incontinence. Persistent or recurrent urinary tract infections may also signal underlying renal complications.^[4]^

##### Hypertension and fluid balance.

Hypertension, often associated with renal involvement, may manifest in MS patients. Fluid balance disturbances, influenced by neurogenic bladder dysfunction, can contribute to hypertension and impact renal function.^[5]^

##### Edema and fluid retention.

Renal dysfunction in MS may lead to fluid retention and edema, presenting as peripheral swelling. This clinical manifestation highlights the need for a thorough examination of fluid status in individuals with both MS and renal concerns.^[3]^

##### Elevated blood pressure.

Elevated blood pressure may serve as an early indicator of renal involvement. Regular blood pressure monitoring is crucial in the clinical evaluation of MS patients, particularly when assessing the potential impact of renal complications.^[6]^

#### 5.2.2. Diagnostic strategies

##### Routine renal function tests

Comprehensive renal function tests, including serum creatinine, blood urea nitrogen, and estimated GFR, form the foundation of renal assessment. Routine monitoring helps detect subtle changes in renal function.^[7]^

##### Urinalysis and microalbuminuria

Urinalysis aids in identifying abnormalities such as hematuria or proteinuria. Detection of microalbuminuria can indicate early renal dysfunction, emphasizing the importance of regular urine examinations.^[9]^

##### Imaging studies

Imaging studies, including renal ultrasound or computed tomography scans, may be employed to assess structural abnormalities, detect hydronephrosis, or identify renal calculi. These studies contribute to a comprehensive evaluation of the renal system.^[10]^

##### Blood pressure monitoring

Regular blood pressure monitoring is essential in MS patients to detect hypertension, a common feature associated with renal complications. Consistent elevation in blood pressure may prompt further investigation into renal function.^[11]^

##### Neurological evaluation

Considering the intertwined nature of MS and renal dysfunction, a neurological evaluation is essential. Assessing the severity of neurogenic bladder dysfunction and its impact on renal function contributes to a holistic understanding of the clinical presentation.^[13]^

##### Specialized renal studies

In specific cases, specialized renal studies such as renal biopsies or nuclear medicine scans may be warranted for a detailed assessment of renal structure and function. These studies are beneficial when the clinical presentation suggests complex or atypical renal involvement.^[6]^

### 5.3. Treatment and management of renal disease in MS patients

The treatment and management of renal disease in patients with MS necessitate a comprehensive and multidisciplinary approach, considering the intricate interplay between neurological and renal health. Optimal care requires addressing specific renal conditions, mitigating factors contributing to renal dysfunction, and tailoring interventions to individual patient needs.

1. Management of neurogenic bladder dysfunction

Initiating effective management strategies for neurogenic bladder dysfunction is paramount. This may include intermittent catheterization, pharmacotherapy to improve detrusor-sphincter coordination and lifestyle modifications. Close collaboration between urologists and neurologists is crucial for optimizing bladder function and preventing complications.^[10]^

2. Blood pressure control

Rigorous blood pressure control is imperative to slow the progression of renal disease. Antihypertensive agents, such as angiotensin-converting enzyme inhibitors (inhibitors) or angiotensin II receptor blockers, are often considered for their renoprotective effects. Regular blood pressure monitoring and titration of medications contribute to maintaining optimal renal perfusion.^[11]^

3. Immunomodulatory therapy adjustment

When MS patients with renal involvement receive immunomodulatory therapies, careful consideration is given to the potential impact on renal function. Treatment plan adjustments may be necessary, including dosages or the choice of therapeutic agents. This necessitates close collaboration between neurologists and nephrologists to balance managing both conditions.^[12]^

4. Treatment of underlying renal conditions

Tailoring treatment to address the specific underlying renal conditions identified through diagnostic assessments is crucial. For example, immunosuppressive therapies may be indicated in autoimmune renal diseases, while interventions like diuretics or surgical measures may be considered for cases involving obstructive uropathy.^[15]^

5. Lifestyle modifications

Encouraging lifestyle modifications, including dietary adjustments, physical activity, and weight management, contributes to overall renal health. These modifications are particularly relevant in the context of metabolic syndrome, a condition that may coexist with MS and impact renal function.^[16]^

6. Monitoring and surveillance

Establishing a systematic approach to monitoring and surveillance is essential. Regular renal function tests, urinalysis, and imaging studies facilitate early detection of changes, enabling prompt intervention. The frequency and scope of monitoring are tailored based on the specific renal condition and the individual overall health status.^[17]^

7. Nutritional counseling

Nutritional counseling, especially in the context of metabolic syndrome, is valuable. Dietary modifications aimed at reducing sodium intake, managing cholesterol levels, and promoting a kidney-friendly diet contribute to managing renal disease in MS patients.^[18]^

8. Patient education and support

Providing comprehensive education to patients about the interaction between MS and renal health is essential. Empowering individuals with knowledge about their conditions, treatment strategies, and the importance of adherence fosters active participation in their care. Support groups and resources may further enhance patient engagement and coping mechanisms.^[19]^

9. Multidisciplinary collaboration

Collaboration between a multidisciplinary team, including neurologists, nephrologists, urologists, dietitians, and other healthcare professionals, is fundamental. Regular interdisciplinary meetings facilitate ongoing communication, ensuring a cohesive and integrated approach to managing renal disease in MS patients.^[20]^

### 5.4. Prognosis of renal diseases in patients with MS

The prognosis of renal diseases in patients with MS represents a complex and multifaceted landscape characterized by interactions between the underlying renal condition, MS itself, and the influence of various therapeutic interventions.^[20]^ Extensive research has been dedicated to understanding the long-term outcomes and prognostic factors associated with renal complications in MS.

1. Impact of MS on renal prognosis

Studies suggest that the presence of MS may influence the prognosis of renal diseases, with the chronic inflammatory state associated with MS potentially contributing to the progression of renal pathology.^[21]^ The systemic effects of MS, including immune dysregulation and neurogenic factors, may burden renal function. However, the impact varies depending on the type and severity of the renal condition.

2. Renal involvement in MS subtypes

The diverse clinical presentations of MS, including relapsing-remitting, secondary progressive, and primary progressive subtypes, add complexity to the prognostic landscape.^[22]^ Limited data indicate that individuals with progressive forms of MS may be at a higher risk of developing renal complications, emphasizing the need for personalized risk stratification based on the MS subtype.^[23]^

3. Immunomodulatory therapies and renal prognosis

The widespread use of immunomodulatory therapies in MS management introduces an additional layer of complexity to the prognosis of renal diseases. While these therapies aim to modulate the immune response within the central nervous system, their systemic effects may influence renal function.^[24]^ Longitudinal studies assessing the impact of various MS treatments on renal outcomes are crucial for understanding the nuanced relationship between therapy and prognosis.

4. Renal-specific prognostic factors

Prognostic factors specific to renal diseases in individuals with MS include the type of renal condition (glomerular dysfunction, tubulointerstitial nephritis, autoimmune renal diseases, etc), severity at the time of diagnosis, and comorbidities such as hypertension and diabetes. Identifying these factors allows for a more accurate assessment of the overall renal prognosis.^[25]^

5. Impact of neurogenic bladder dysfunction

Individuals with MS often experience neurogenic bladder dysfunction, which can contribute to renal complications. The prognosis of renal outcomes may be influenced by the severity of bladder dysfunction, the effectiveness of bladder management strategies, and the prevention of complications such as urinary tract infections and hydronephrosis.^[26]^

6. Long-term monitoring and surveillance

Proactive and regular monitoring of renal function through laboratory assessments, imaging studies, and clinical evaluations is essential for a comprehensive understanding of prognosis. Long-term surveillance allows for the early detection of renal complications, enabling timely interventions and improved outcomes.^[30]^

7. Interdisciplinary care and management

Collaborative care involving neurologists, nephrologists, urologists, and other healthcare providers is crucial for optimizing the prognosis of renal diseases in individuals with MS. Integrated management plans that address both neurological and renal aspects contribute to a holistic approach to patient care.^[31]^

### 5.5. Impact of renal disease on QOL in MS patients

Renal disease can significantly impact the overall QOL for individuals with MS, and patient perspectives have highlighted several key aspects of this impact.

#### 5.5.1. Physical health

Renal disease often leads to physical symptoms such as fatigue, fluid retention, and changes in blood pressure. These symptoms can exacerbate the physical challenges already faced by individuals with MS, further reducing their mobility and energy levels.^[1]^

#### 5.5.2. Mental health

The added burden of renal disease can increase stress and anxiety for MS patients. Coping with multiple chronic conditions and managing treatment regimens can affect mental well-being.^[2]^

#### 5.5.3. Treatment burden

Managing renal disease alongside MS requires adherence to multiple medications, dietary restrictions, and regular medical appointments. This treatment burden can be overwhelming and affect daily routines.^[3]^

#### 5.5.4. Social and emotional impact

Renal disease may limit social activities and dietary choices, impacting an individual ability to participate in social events and enjoy their favorite foods. This can lead to feelings of isolation and frustration.^[4]^

#### 5.5.5. Financial strain

The cost of medical treatments, medications, and frequent doctor visits can place a significant financial strain on individuals with MS and renal disease. This can impact their ability to access necessary care and maintain their overall QOL.^[5]^

#### 5.5.6. Patient perspectives

Patient perspectives on living with both MS and renal disease emphasize the need for a holistic approach to healthcare. MS patients emphasize the importance of communication between their healthcare providers to coordinate care and manage comorbidities effectively. They also stress the need for patient education and support to help them navigate the challenges of living with both conditions.^[6]^

The impact of renal disease on the QOL of individuals with MS underscores the importance of comprehensive care that addresses both conditions and considers the individual physical, mental, and emotional well-being. Healthcare providers must communicate openly with patients, involve them in treatment decisions, and provide resources and support to enhance their QOL while managing these complex health issues.

### 5.6. Future directions and research gaps in renal disease and MS

While there has been growing interest in understanding the relationship between renal disease and MS, several research gaps exist, and further investigations are needed to advance our knowledge in this area. Here are some potential directions for future studies:

#### 5.6.1. Longitudinal studies

Conduct long-term prospective studies to examine the natural progression of renal disease in MS patients. This would provide insights into the temporal relationship between MS and renal disease development and help identify risk factors associated with their co-occurrence.^[1]^

#### 5.6.2. Mechanistic studies

Investigate the underlying mechanisms linking MS and renal disease. Explore how immune dysregulation, genetic factors, and chronic inflammation contribute to the development and progression of both conditions. Understanding these mechanisms could lead to targeted therapies.^[2]^

#### 5.6.3. Impact on treatment

Assess the effect of renal disease on the choice and efficacy of DMTs for MS. Investigate potential interactions between DMTs and renal function, as well as the safety and effectiveness of specific DMTs in MS patients with renal disease.^[3]^

#### 5.6.4. Patient-centered research

Include patient perspectives in research design and outcomes assessment. Understand how renal disease affects MS patients’ QOL, treatment preferences, and adherence to therapy. Develop interventions tailored to patient needs.^[4]^

#### 5.6.5. Biomarkers

Identify biomarkers that can predict the development of renal disease in MS patients or serve as indicators of renal disease severity. Biomarkers could aid in early detection and monitoring.^[5]^

#### 5.6.6. Intervention trials

Conduct clinical trials to evaluate the effectiveness of interventions to prevent or manage renal disease in MS patients. These interventions might include lifestyle modifications, early screening, or novel therapeutic approaches.^[6]^

#### 5.6.7. Epidemiological studies

Conduct large-scale epidemiological studies to determine the prevalence of renal disease in MS populations across different regions and ethnicities. Identify geographical and demographic factors associated with the co-occurrence of these conditions.^[7]^

#### 5.6.8. Precision medicine

Explore the potential for precision medicine approaches in managing MS patients with renal disease. Personalized treatment plans considering individual genetics and disease characteristics may optimize outcomes.^[8]^

#### 5.6.9. Comorbidities management

Investigate strategies for integrated care that address MS and renal disease and other comorbidities commonly seen in MS patients, such as diabetes and cardiovascular disease.^[9]^

#### 5.6.10. Healthcare delivery models

Examine healthcare delivery models that facilitate coordinated care for individuals with MS and renal disease. Evaluate the impact of multidisciplinary teams on patient outcomes and QOL.^[10]^

Future research efforts in these areas will contribute to a deeper understanding of the complex interplay between MS and renal disease, ultimately leading to improved care, earlier detection, and more effective management strategies for individuals with both conditions.

## 6. Conclusion

In this narrative review, we have explored the intricate relationship between renal disease and MS, shedding light on the coexistence of these conditions and their potential implications for affected individuals. Key findings and insights from this review underscore several critical points:

### 6.1. Prevalence and complexity

Renal disease is not uncommon in the MS population, with evidence suggesting that individuals with MS may be at a higher risk of developing renal abnormalities such as proteinuria and decreased GFR. The coexistence of these conditions presents a complex clinical scenario.

### 6.2. Shared mechanisms

While MS and renal disease primarily affect different organ systems, there is growing evidence of shared immunological and inflammatory mechanisms that may contribute to their co-occurrence. Immune dysregulation, genetic factors, and chronic inflammation are potential common denominators linking these conditions.

### 6.3. Impact on QOL

Renal disease in MS patients can profoundly affect their overall QOL. The physical, mental, and emotional toll of managing both conditions, treatment burdens, and potential financial strain highlights the need for comprehensive, patient-centered care.

### 6.4. Research gaps

The review has also identified several research gaps, including the need for longitudinal studies, mechanistic investigations, and clinical trials tailored to MS patients with renal disease. Future research should unravel the underlying mechanisms, optimize treatment strategies, and improve healthcare delivery for these individuals.

Understanding the intricate interplay between MS and renal disease is of paramount importance. This understanding can lead to earlier detection, more effective management, and improved overall outcomes for individuals facing the dual challenge of these chronic conditions. It emphasizes the significance of a holistic healthcare approach considering the neurological and renal aspects of patients’ well-being.

Finally, this narrative review is a foundation for further research and clinical practice in MS and renal disease. By addressing the knowledge gaps and fostering collaboration among healthcare providers and researchers, we can strive to enhance the lives of individuals living with both MS and renal disease, ultimately improving their prognosis and QOL.

## Author contributions

**Conceptualization:** Chukwuka Elendu, Dependable C. Amaechi, Tochi C. Elendu, Michael O. Adegbola.

**Data curation:** Chukwuka Elendu, Dependable C. Amaechi, Tochi C. Elendu.

**Formal analysis:** Chukwuka Elendu, Dependable C. Amaechi, Tochi C. Elendu, Mutalib O. Ozigi, Michael O. Adegbola.

**Funding acquisition:** Chukwuka Elendu, Dependable C. Amaechi, Tochi C. Elendu, Mutalib O. Ozigi, Michael O. Adegbola, Mololuwa A. Adebayo.

**Investigation:** Chukwuka Elendu, Dependable C. Amaechi, Tochi C. Elendu, Oluwatosin G. Afolabi.

**Methodology:** Chukwuka Elendu, Dependable C. Amaechi, Tochi C. Elendu, Mutalib O. Ozigi, Oluwatosin G. Afolabi.

**Project administration:** Chukwuka Elendu, Dependable C. Amaechi, Tochi C. Elendu, Mololuwa A. Adebayo.

**Resources:** Chukwuka Elendu, Dependable C. Amaechi, Tochi C. Elendu, Michael O. Adegbola.

**Software:** Chukwuka Elendu, Dependable C. Amaechi, Tochi C. Elendu, Mutalib O. Ozigi, Oluwatosin G. Afolabi.

**Supervision:** Chukwuka Elendu, Dependable C. Amaechi, Tochi C. Elendu, Mololuwa A. Adebayo.

**Validation:** Chukwuka Elendu, Dependable C. Amaechi, Tochi C. Elendu, Mutalib O. Ozigi.

**Visualization:** Chukwuka Elendu, Dependable C. Amaechi, Tochi C. Elendu, Mololuwa A. Adebayo.

**Writing – original draft:** Chukwuka Elendu, Dependable C. Amaechi, Tochi C. Elendu

**Writing – review & editing:** Chukwuka Elendu, Dependable C. Amaechi, Tochi C. Elendu, Mutalib O. Ozigi, Michael O. Adegbola, Oluwatosin G. Afolabi

## References

[1] Compston A Coles A. Multiple sclerosis. Lancet. 2008;372:1502–17. 18970977 10.1016/S0140-6736(08)61620-7

[2] Chwastiak LA Ehde DM. Psychiatric issues in multiple sclerosis. Psychiatr Clin North Am. 2007;30:803–17. 17938046 10.1016/j.psc.2007.07.003 PMC2706287

[3] Calabresi PA Austin H Racke MK. Impaired renal function in progressive multiple sclerosis. Neurology. 2002;59:1799–801. 12473777 10.1212/01.wnl.0000036618.68674.7a

[4] Macor S Porrini AM Giampietro A. Multiple sclerosis: an immune system activation disease. Acta Neurol (Napoli). 1991;13:590–6. 1839591

[5] Alugba G Urhi A Olateju IV. Renal diseases associated with multiple sclerosis: a narrative review. Medicine (Baltim). 2022;101:e31959. 10.1097/MD.0000000000031959 PMC9726425 36482579

[6] Marrie RA Cohen J Stuve O. A systematic review of the incidence and prevalence of comorbidity in multiple sclerosis: overview. Mult Scler. 2015;21:263–81. 25623244 10.1177/1352458514564491 PMC4361468

[7] Hauser SL Oksenberg JR. The neurobiology of multiple sclerosis: genes, inflammation, and neurodegeneration. Neuron. 2006;52:61–76. 17015227 10.1016/j.neuron.2006.09.011

[8] Trapp BD Nave KA. Multiple sclerosis: an immune or neurodegenerative disorder? Annu Rev Neurosci. 2008;31:247–69. 18558855 10.1146/annurev.neuro.30.051606.094313

[9] Bjartmar C Trapp BD. Axonal and neuronal degeneration in multiple sclerosis: mechanisms and functional consequences. Curr Opin Neurol. 2001;14:271–8. 11371748 10.1097/00019052-200106000-00003

[10] Lassmann H van Horssen J Mahad D. Progressive multiple sclerosis: pathology and pathogenesis. Nat Rev Neurol. 2012;8:647–56. 23007702 10.1038/nrneurol.2012.168

[11] Alvarez JI Cayrol R Prat A. Disruption of central nervous system barriers in multiple sclerosis. Biochim Biophys Acta. 2011;1812:252–64. 20619340 10.1016/j.bbadis.2010.06.017

[12] Franklin RJ Ffrench-Constant C. Remyelination in the CNS: from biology to therapy. Nat Rev Neurosci. 2008;9:839–55. 18931697 10.1038/nrn2480

[13] Ascherio A Munger KL. Environmental risk factors for multiple sclerosis. Part I: the role of infection. Ann Neurol. 2007;61:288–99. 17444504 10.1002/ana.21117

[14] Levey AS Eckardt KU Tsukamoto Y. Definition and classification of chronic kidney disease: a position statement from kidney disease: improving global outcomes (KDIGO). Kidney Int. 2005;67:2089–100. 15882252 10.1111/j.1523-1755.2005.00365.x

[15] Jha V Garcia-Garcia G Iseki K. Chronic kidney disease: global dimension and perspectives. Lancet. 2013;382:260–72. 23727169 10.1016/S0140-6736(13)60687-X

[16] Lameire NH Bagga A Cruz D. Acute kidney injury: an increasing global concern. Lancet. 2013;382:170–9. 23727171 10.1016/S0140-6736(13)60647-9

[17] Wyatt CM Klotman PE D’Agati VD. HIV-associated nephropathy: clinical presentation, pathology, and epidemiology in the era of antiretroviral therapy. Semin Nephrol. 2008;28:513–22. 19013322 10.1016/j.semnephrol.2008.08.005 PMC2656916

[18] Torres VE Harris PC Pirson Y. Autosomal dominant polycystic kidney disease. Lancet. 2007;369:1287–301. 17434405 10.1016/S0140-6736(07)60601-1

[19] Tuttle KR Bakris GL Bilous RW. Diabetic kidney disease: a report from an ADA Consensus Conference. Diabetes Care. 2014;37:2864–83. 25249672 10.2337/dc14-1296 PMC4170131

[20] Treacy O Brown NN Dimeski G. Biochemical evaluation of kidney disease. Transl Androl Urol. 2019;8(Suppl 2):S214–23. 31236339 10.21037/tau.2018.10.02 PMC6559936

[21] Fox RJ Miller DH Phillips JT. Placebo-controlled phase 3 study of oral BG-12 or glatiramer in multiple sclerosis. N Engl J Med. 2012;367:1087–97. 22992072 10.1056/NEJMoa1206328

[22] Shahrokhi M Asuncion RMD. Neurologic Exam. [Updated 2023 Jan 16]. In: StatPearls. Treasure Island (FL): StatPearls Publishing. 2023. Available at: https://www.ncbi.nlm.nih.gov/books/NBK557589/. 32491521

[23] Wu GF Alvarez E. The immunopathophysiology of multiple sclerosis. Neurol Clin. 2011;29:257–78. 21439440 10.1016/j.ncl.2010.12.009 PMC3080042

[24] Baranzini SE Oksenberg JR. The genetics of multiple sclerosis: from 0 to 200 in 50 years. Trends Genet. 2017;33:960–70. 28987266 10.1016/j.tig.2017.09.004 PMC5701819

[25] Libby P Ridker PM Maseri A. Inflammation and atherosclerosis. Circulation. 2002;105:1135–43. 11877368 10.1161/hc0902.104353

[26] Beishon L Haunton VJ Panerai RB. Cerebral hemodynamics in mild cognitive impairment: a systematic review. J Alzheimers Dis. 2017;59:369–85. 28671118 10.3233/JAD-170181

[27] Colonna L Lood C Elkon KB. Beyond apoptosis in lupus. Curr Opin Rheumatol. 2014;26:459–66. 25036095 10.1097/BOR.0000000000000083 PMC4272326

[28] Cameron JS Hicks J. The origins and development of the concept of a “nephrotic syndrome. Am J Nephrol. 2002;22:240–7. 12097747 10.1159/000063768

[29] Bakris GL. Recognition, pathogenesis, and treatment of different stages of nephropathy in patients with type 2 diabetes mellitus. Mayo Clin Proc. 2011;86:444–56. 21531886 10.4065/mcp.2010.0713 PMC3084647

[30] Alicic RZ Rooney MT Tuttle KR. Diabetic kidney disease: challenges, progress, and possibilities. Clin J Am Soc Nephrol. 2017;12:2032–45. 28522654 10.2215/CJN.11491116 PMC5718284

[31] Khwaja A. KDIGO clinical practice guidelines for acute kidney injury. Nephron Clin Pract. 2012;120:c179–84. 22890468 10.1159/000339789

